# Treatment of refractory juvenile idiopathic arthritis via pulse therapy using methylprednisolone and cyclophosphamide

**DOI:** 10.1590/S1516-31802003000300006

**Published:** 2003-05-01

**Authors:** Tania Caroline Monteiro de Castro, Maria Teresa Terreri, Claudio Len, Maria Odete Esteves Hilário

**Keywords:** Rheumatoid athritis, Juvenile rheumatoid arthritis, Arthritis, Methylprednisolone, Cyclophosphamide, Methotrexate, Artrite reumatóide, Artrite reumatóide Juvenil, Artrite, Metilprednisolona, Ciclofosfamida, Metotrexate

## Abstract

**CONTEXT:**

Patients with refractory juvenile idiopathic arthritis can benefit from aggressive therapy.

**CASE REPORT:**

We followed the clinical course of 4 patients (2 male, 2 female) aged 9.1-17.8 years (mean of 14.5 years) with polyarticular onset of juvenile rheumatoid arthritis and one 16-year-old boy with juvenile spondyloarthropathy associated with inflammatory bowel disease. All the juvenile rheumatoid arthritis patients fulfilled the diagnostic criteria established by the American College of Rheumatology. All patients had unremitting arthritis despite maximum therapy. All patients began receiving treatment using intravenous cyclophosphamide at 500-750 mg/m and intravenous methylprednisolone at 30 mg/kg, for 3 days monthly (1 g maximum). The patients received between 3 and 11 monthly treatments, and/or 3-5 treatments every two months for 12 months, according to the severity of the disease and/or response to the therapy. All but one patient were evaluated retrospectively at the start (time 0) and 6 months (time 1), and 12 months (time 2) after the beginning of the treatment. A rapid and clinically significant suppression of systemic and articular manifestations was seen in all patients. Our results showed the favorable effect of this treatment on the clinical and some laboratory manifestations of juvenile idiopathic arthritis.

## RESUMO

**CONTEXTO:**

Pacientes com artrite idiopática juvenil refratária podem se beneficiar com uma terapia mais agressiva.

**RELATO DE CASO:**

A artrite reumatóide juvenil é a causa mais comum de artrite crônica em crianças definida como artrite por mais de seis semanas em pacientes com idade igual ou inferior a 16 anos. Ela é considerada refratária quando não responde ao tratamento com metotrexato em doses elevadas (1 mg/kg/semana subcutâneo). Nesses casos, terapias de associação de medicamentos ou terapias mais agressivas, como pulso de solumedrol e ciclofosfamida podem ser indicadas. Quatro pacientes com artrite reumatóide juvenil e um com espondiloartropatia juvenil associada a doença inflamatória intestinal foram acompanhados em nosso serviço. Todos receberam pulsoterapia com ciclofosfamida intravenosa (500-750 mg/m^2^) e metilprednisolona intravenosa (30 mg/kg/dia). Todos os pacientes receberam entre 3 e 11 pulsos mensais e/ou 3 a 5 pulsos a cada 2 meses por um período de 12 meses de acordo com a gravidade da doença e/ou a resposta à terapia. Todos, exceto um paciente, foram avaliados retrospectivamente no início (tempo 0), 6 meses (tempo 1) e 12 meses (tempo 2) após iniciar o tratamento. Uma rápida e significante supressão das manifestações articulares e sistêmicas foi observada em todos os pacientes. Nossos resultados mostraram um efeito favorável deste esquema terapêutico nas manifestações clínicas e laboratoriais da artrite idiopática juvenil. A redução da dose diária de corticóide oral deve ser considerada um dos mais importantes efeitos nestes pacientes.

## INTRODUCTION

Juvenile rheumatoid arthritis is the most common cause of chronic arthritis in children. It is defined as an arthritis complaint lasting 6 weeks or longer in patients 16 years of age and younger that has no known etiology. This is a diagnosis of exclusion, so other causes of chronic arthritis must be ruled out.^1,2^

Juvenile rheumatoid arthritis has been divided into subtypes, but we are still unable to identify those children who will progress to poor outcomes and might benefit from more aggressive therapy.^3^ Many children with juvenile rheumatoid arthritis respond to a limited course of nonsteroidal anti-inflammatory drugs, especially those with oligoarticular onset. Sometimes, these drugs associated with intra-articular corticosteroid are often the only treatment needed.^4^ Most patients with systemic or polyarticular onset need second-line medication. Hydroxychloroquine or methotrexate is useful as an adjunct to nonsteroidal anti-inflammatory drugs. Glucocorticoids have been tried with good results in juvenile rheumatoid arthritis, but their side effect profile precludes routine use. They should be used for shorter periods of time. Immunosuppressive drugs are being increasingly utilized early on in patients with juvenile rheumatoid arthritis who need aggressive therapy.

Refractory juvenile rheumatoid arthritis should be considered when the disease does not respond to high doses of methotrexate (1 mg/kg/week subcutaneously). In such cases, therapies involving combinations of drugs or more aggressive therapies like intravenous methylprednisolone and cyclophosphamide can be considered, since the use of biological agents is not possible for most patients.

Our purpose was to clinically and sero-logically evaluate the treatment outcomes from four patients with a severe form of juvenile rheumatoid arthritis and one with juvenile spondyloarthropathy, treated using intravenous pulses of cyclophosphamide and methylprednisolone and high doses of subcutaneous methotrexate over periods ranging from 8 to 11 months.

## CASE REPORT

### Patient 1

A 9.1-year-old boy had developed rash, fever and polyarthritis in the large joints of the cervical spine, shoulders, wrists and ankles at the age of 2.6 years. He presented persistent anemia, leukocytosis and thrombocytosis, and an elevated erythrocyte sedimentation rate, despite therapy using prednisone, nonsteroidal anti-inflammatory drugs and methotrexate. He maintained fever, rash and arthritis when we started a course of immunoglobulin IV monthly, with cyclosporin and pulse therapy using methylprednisolone, with no improvement. Because of persistent active disease, we started pulses of intravenous methylprednisolone with cyclophosphamide (11 monthly treatments). The patient showed good response with improvement in the clinical and laboratory parameters.

### Patient 2

A 14.3-year-old girl had started fever and polyarthritis at the age of 2 years. She presented small joint disease involving the hands and feet.

The cervical spine, shoulders, elbows, wrists, hips, knees and ankles were also involved. She presented anemia, leukocytosis, thrombocytosis and an elevated erythrocyte sedimentation rate. She used prednisone, nonsteroidal anti-inflammatory drugs, immunoglobulin IV monthly and methotrexate, with no clinical and laboratory improvement. She developed toxicity with elevation of hepatic enzyme levels, and methotrexate was discontinued. Cyclosporin was introduced but the patient persisted with disease activity. We started pulses of intravenous methylprednisolone and cyclophosphamide (3 monthly and 3 bimonthly treatments). The patient showed improvement in the clinical and laboratory activity.

### Patient 3

This 15.5-year-old girl had started polyarticular onset disease at the age of 1.4 years. She presented fever and rash, with small and large joint disease involving the hands, feet, shoulders, elbows, wrists, hips and knees. She had unremitting disease despite therapy with prednisone, nonsteroidal anti-inflammatory drugs, methotrexate, azathioprine and pulses of methylprednisolone. She was treated using pulse therapy of intravenous methylprednisolone with cyclophosphamide (5 bimonthly treatments), with improvement in her joint symptoms and laboratory results.

### Patient 4

This 17.8-year-old boy had presented with polyarthritis in the large joints of the cervical spine, shoulders, elbows, wrists, hips, knees and ankles at the age of 7 years. He had not obtained improvement with prednisone, non-steroidal anti-inflammatory drugs, methotrexate, hydroxychloroquine and pulses of methylprednisolone. He started a course of azathioprine because of his unremitting disease. The patient showed improvement after treatment using pulse therapy of intravenous methylprednisolone with cyclophosphamide (5 bimonthly treatments).

### Patient 5

A 16-year-old boy had developed spondyloarthropathy associated with inflammatory bowel disease at the age of 2 years. He presented involvement of elbows, wrists and knees. He was initially treated with prednisone, nonsteroidal anti-inflammatory drugs and methotrexate and then with sulfasalazine and azathioprine, with no improvement. He persisted with active disease until the beginning of pulse therapy of intravenous methylprednisolone with cyclophosphamide (8 monthly treatments). He showed improvement in the number of joints with active disease and the erythrocyte sedimentation rate.

## RESULTS

Characteristics of the five patients are shown in Table 1. Disease onset occurred between the ages of 1.4 and 7.0 years (mean of 3.0 years) and patient ages at the time of the study were 9.1 to 17.8 years (mean of 14.5 years). Disease duration was considered to be the period of time from the first clinical manifestations until the beginning of the therapy (methylprednisolone, cyclophosphamide and methotrexate). It ranged from 6.5 to 14.1 years (mean of 11.5 years).

**Table 1:** Clinical and epidemiological characteristics, medications in use and treatment frequency of 5 patients with juvenile rheumatoid arthritis and juvenile spondyloarthropathy

	Patient 1	Patient 2	Patient 3	Patient 4	Patient 5
Sex	M	F	F	M	M
Age, in years	9.1	14.3	15.5	17.8	16.0
JRA subtype	polyarticular	polyarticular	polyarticular	polyarticular	-
Age at onset, years	2.6	2.0	1.4	7.0	2.0
Duration of disease, in years	6.5	12.3	14.1	10.8	14.0
Medications in use	cyclosporin 2.8 mg/kg/day MTX 0.9 mg/kg/week; immunoglobulin 2 g/kg	cyclosporin 4.5 mg/kg/day	azathioprine 2.2 m/kg/day; MTX 0.9 mg/kg/week	azathioprine 2.0 m/kg/day; MTX 0.9 mg/kg/week	sulfasalazine 30 mg/kg/day; MTX 1 mg/kg/week
Pulses	CYC and MP IV monthly, for 11 months	CYC and MP IV monthly, for 3 months, and bimonthly for 3 months	CYC and MP IV bimonthly for 5 months	CYC and MP IV bimonthly for 5 months	CYC and MP IV monthly, for 8 months
**Total of pulses**	**11**	**6**	**5**	**5**	**8**

*JRA = juvenile rheumatoid arthritis; MTX = methotrexate; M = male; F = female; IV = intravenous; CYC = cyclophosphamide; MP = methylprednisolone.*

All the juvenile rheumatoid arthritis patients presented polyarticular onset. Each patient received between 3 and 11 monthly treatments of intravenous methylprednisolone and cyclophosphamide, and 3-5 intravenous methylprednisolone and cyclophosphamide infusions every two months for 12 months (Table 1). Clinical and serological improvement was noted in four patients (Table 2). The number of joints with active disease was reduced in four patients, as was the erythrocyte sedimentation rate in four patients. Hemoglobin levels increased in four patients. All patients required lower doses of corticosteroids and two discontinued this medication (Table 2). During the evaluation period, only one patient underwent modification of the therapy (azathioprine use), with no improvement. Two patients took cyclosporin, two patients azathioprine, one patient intravenous immunoglobulin and one patient sulfasalazine over the whole follow-up period (Table 1). With regard to side effects, patient 4 presented nausea and patient 1 hypertension. Improvement was noted in both patients after symptomatic therapy. No patients in this study went into remission. All subjects were at or below the 10^th^ percentile in height for age.

**Table 2:** Clinical and laboratory characteristics, and corticosteroid doses at the start and 6 months and 1 year after the start of treatment (cyclophosphamide + methylprednisolone + methotrexate), of 5 patients with juvenile rheumatoid arthritis and juvenile spondyloarthropathy

	Patient 1			Patient 2			Patient 3			Patient 4			Patient 5		
Months after start of treatment	0	6	12	0	6	12	0	6	12	0	6	12	0	6	12
Number of active joints	1	1	2	8	2	1	5	1	1	3	7	2	5	3	3
Steinbrocker functional class	II		I	IV		II	IV		II	III		II	II		II
Hb, g/100 ml	9.8	12.7	13.0	7.3	9.8	9.7	10.1	9.6	10.2	12.8	8.2	9.2	8.2	9.7	8.8
Htc, %	31	40.0	39.7	24	30	31.4	34	32	31	41	28	29	27	32	30.5
WBC, x1000/cm^2^	9.7	11.3	6.4	15.0	16.4	10.8	9.8	4.4	6.9	11.3	12.4	11.0	5.5	5.4	3.5
Platelets, x1000/cm^2^	117	442	418	762	704	599	519	290	352	403	709	303	240	280	241
ESR, mm/hour	71	22	22	125	91	60	40	4	48	57	26	45	54	12	14
CS, mg/kg/day	0.28	0.17	0.14	0.41	0.15	0	0.21	0.20	0.08	0.34	0.17	0.09	0.20	0	0

*Hb = hemoglobin; Htc = hematocrit; WBC = white blood cell count; ESR = Westergren erythrocyte sedimentation rate; CS = corticosteroids.*

## DISCUSSION

The aim in treating juvenile rheumatoid arthritis is to minimize discomfort, reduce disability, prevent joint destruction, and maximize physical, social and emotional development. However, successful management of severe juvenile rheumatoid arthritis remains a challenge in pediatric rheumatology.^5^

Pulse therapy using methylprednisolone is an alternative to oral corticosteroids, in order to reduce the side effects. Job-Deslandre and Menkes^6^ retrospectively evaluated the results from methylprednisolone pulses in 15 children with chronic arthritis (13 cases of juvenile rheumatoid arthritis and two cases of spondyloarthropathy). Methylprednisolone succinate was administered at a dosage of 700 mg/m^2^ by an intravenous infusion pump on 3 consecutive days. A dramatic clinical improvement was obtained in 12/15 cases by day 4. In seven cases, multiple pulses (between two and eight courses) were administered to obtain better control of the disease and a decrease in the daily dosage of corticosteroids. The authors observed only mild and transient side effects and a decrease in the previous side effects from the corticosteroids (especially growth retardation).

Azathioprine and cyclophosphamide have been used in the treatment of juvenile rheumatoid arthritis but their potential toxicity is greater. The benefits from pulse cyclophosphamide in comparison with oral cyclophosphamide include reduction in the total dose and the risk of side effects. Adequate hydration can be provided, so as to protect the bladder from hemorrhagic cystitis.^7^ Other "disease-modifying therapies" that are being tried in juvenile rheumatoid arthritis include hydroxychloroquine, sulfasalazine, intravenous immunoglobulin, cyclosporin, and combination therapy .^3,8,9^ The most commonly used disease-modifying antirheumatic drug presently used in juvenile rheumatoid arthritis is methotrexate, particularly in polyarticular and systemic juvenile rheumatoid arthritis. It has been shown to be safe and effective in retrospective studies, clinical practice and controlled trials.^10,11^ Serious long-term toxicity appears to be infrequent.^10,11^

Pulse cyclophosphamide with methylprednisolone has been proposed for the treatment of severe systemic-onset juvenile rheumatoid arthritis.^12^ Wallace and Sherry^13^ studied four children (two male, two female) with systemic-onset juvenile rheumatoid arthritis, joint destruction and polyarthritis that remained active despite maximal therapy with combination of drugs. Intravenous cyclophosphamide (500-1000 mg/m^2^) and methylprednisolone 30 mg/kg/day (1 g maximum) were given monthly. Patients received six to ten monthly treatments followed by two to thirteen subsequent treatments every two to three months. All patients showed clinical improvement with 12-20 intravenous pulses. Three patients achieved disease remission despite the discontinuation of cyclophosphamide. Shaikov et al.^14^ evaluated the effectiveness of pulse therapy consisting of methylprednisolone 30 mg/kg/day for three consecutive days, cyclophosphamide 400 mg/m^2^ and methotrexate 10 mg/m^2^ in 18 patients (10 male, 8 female) with systemic juvenile rheumatoid arthritis. The children received pulse therapy every three months in an open trial of 12 months duration. A rapid and clinically significant suppression of systemic and articular manifestations was seen in all patients. A significant decrease in laboratory indices of disease activity was also observed. These studies and ours suggest that the addition of pulse therapy of cyclophosphamide and methylprednisolone to the methotrexate treatment is potentially useful in patients with resistant polyarticular or systemic-onset disease.

In our study, all patients had been using methotrexate prior to this treatment, with no results. It was only when we associated intravenous cyclophosphamide and methylprednisolone that we observed a notable clinical and laboratory improvement. No significant side effects were observed during the study.

## CONCLUSION

Our results showed the favorable effect of this treatment on the clinical and some laboratory manifestations of juvenile idiopathic arthritis. The reduction in the daily dose of oral steroids must be considered one of the most important effects among such patients. These data support the need for controlled trials using cyclophosphamide, methylprednisolone and methotrexate in a larger cohort of patients with juvenile idiopathic arthritis, so as to determine the safety, efficacy, and disease-modifying effects in this inflammatory condition.
